# Influence of Arbuscular Mycorrhizal Fungi on Soybean Growth and Yield: A Metabarcoding Approach

**DOI:** 10.3390/plants15010131

**Published:** 2026-01-02

**Authors:** Wasan Seemakram, Thanapat Suebrasri, Sompong Chankaew, Sophon Boonlue

**Affiliations:** 1 Department of Microbiology and Parasitology, Faculty of Medical Science, Naresuan University, Phitsanulok 65000, Thailand; wasans@nu.ac.th; 2 Faculty of Medical Science, Nakhon Ratchasima College, Nakhon Ratchasima 30000, Thailand; s.thanapat@nmc.ac.th; 3 Department of Agronomy, Faculty of Agriculture, Khon Kaen University, Khon Kaen 40002, Thailand; somchan@kku.ac.th; 4 Plant Breeding Research Center for Sustainable Agriculture, Khon Kaen University, Khon Kaen 40002, Thailand; 5 Department of Microbiology, Faculty of Science, Khon Kaen University, Khon Kaen 40002, Thailand

**Keywords:** *Glycine max* cultivar Morkhor 60, microbiome, organic agriculture, pathogenic fungi, plant growth promotion, rhizosphere

## Abstract

This study evaluated the efficiency of arbuscular mycorrhizal fungi (AMF) in promoting the growth, yield, protein, and phytochemical contents of *Glycine max* cv. Morkhor 60. A completely randomized pot experiment was conducted for 90 days in non-sterile soil with nine replications. Three AMF species were tested and compared with two non-mycorrhizal controls, with and without NPK fertilizer. All AMF treatments enhanced plant growth, photosynthetic rate, and water-use efficiency compared with the unfertilized control. Inoculation with *Acaulospora dilatata* KKU-SK202 produced the highest pod number and increased 100-seed weight by 27.00% and 4.13% over the non-inoculated and NPK treatments, respectively. *Gigaspora margarita* KKU-SK210 yielded the highest total protein and phenolic contents, while *A. dilatata* KKU-SK401 showed the highest antioxidant activity (72.09%). Metabarcoding analysis revealed that AMF inoculation reduced root colonization by pathogenic fungi, with *G. margarita* KKU-SK210 and *A. dilatata* KKU-SK202 being the most effective. These results suggest that AMF inoculation can enhance soybean productivity and seed quality while reducing chemical fertilizer dependency and pathogenic fungal incidence.

## 1. Introduction

Soybean (*Glycine max* (L.) Merr.) is a globally important legume for animal and human consumption as a high-quality protein source [1]. Soybean seed contains approximately 40% protein, 35% soluble sugars/dietary fiber, and 20% oil (20%, which is cholesterol-free and contains both monounsaturated and polyunsaturated fatty acids)). It is a common, rich, healthy, and easily accessible protein source [2]. Additionally, soybean contains a variety of bioactive phytochemicals that provide beneficial effects to the human body. For example, soybean contains isoflavones, tocopherols, soyasaponins, proteins, and oligosaccharides that are effective in various biological activities, such as cholesterol reduction, antioxidant activity, anticarcinogenic, immunostimulatory, and antidiabetic activities, reduction in osteoporosis risk, and reductions in triglyceride levels [3,4]. Accordingly, research on improving plant growth, seed yield, protein content, and functional compounds such as antioxidant and phenol compounds has gained more attention. The most common and easily accessible practice is using synthetic fertilizer to enhance plant production. However, while (over-)use of synthetic fertilizer increases productivity, it may simultaneously result in lower soil quality, notably a reduction in beneficial soil microbes [5]. In order to reduce the use of synthetic fertilizers to promote growth and yield of soybean, arbuscular mycorrhizal fungi (AMF) are of interest that have been extensively studied due to their environmentally friendly nature [6].

AMF are a group of fungi belonging to the Glomeromycota that live in a mutualistic symbiosis with plant roots. These fungi can be found in nearly all ecosystems and 80% of plant species. They develop extraradical mycelia that extend the depletion zone that develops around roots and facilitate the acquisition of nutrients of low mobility [7,8]. In addition to their well-documented role in enhancing plant growth and yield, arbuscular mycorrhizal fungi (AMF) are increasingly recognized for their ability to improve plant tolerance to abiotic stresses, thereby indirectly contributing to plant productivity [9]. AMF colonization has been shown to enhance plant resilience against drought, salinity, and temperature extremes through physiological, biochemical, and molecular mechanisms. Recent transcriptomic studies provide strong evidence that AMF can reprogram plant stress-response pathways under adverse environmental conditions. For example, inoculation of heat-stressed tomato plants with *Septoglomus constrictum* resulted in the differential regulation of genes associated with heat-shock proteins, transporters, reactive oxygen species (ROS)-scavenging enzymes, and hormone signaling pathways, demonstrating a clear AMF-mediated enhancement of thermotolerance [10]. These findings highlight the role of AMF as key modulators of plant stress adaptation and support their potential application in sustainable crop production under changing climatic conditions. They especially have a beneficial effect under unfavorable environmental conditions. Next to increasing plant biomass, they also improve product quality through enhancing the production of secondary compounds, as demonstrated by the increased production of cannabinoids in cannabis (*Cannabis sativa* L.), and anthocyanins, antioxidant, and phenolic compounds in black upland rice (*Oryza sativa* L.) [6,11]. Due to these beneficial features, AMF are good candidates for improving plant productivity and product quality.

Based on previous studies demonstrating the beneficial roles of arbuscular mycorrhizal fungi (AMF) in enhancing plant growth, nutrient uptake, and secondary metabolite production, we hypothesized that inoculation with selected AMF species would improve soybean growth performance, yield, protein accumulation, and the production of antioxidant and phenolic compounds under non-sterile soil conditions. Furthermore, we hypothesized that AMF colonization would influence the diversity and composition of root-associated fungal communities, which may indirectly contribute to plant growth promotion. To test these hypotheses, we employed a controlled pot experiment using three AMF species (*Gigaspora margarita*, *Acaulospora dilatata*, and *A. delicata*). Plant growth, yield components, protein content, and secondary metabolites were quantitatively evaluated, while fungal identification and diversity analyses were performed to characterize root-associated fungal communities. Given the limited information on the effects of arbuscular mycorrhizal fungi (AMF) colonization in soybean roots on other root-associated fungi, we comprehensively identified fungal taxa and their diversity, and investigated their potential on plant growth promotion. The results of this study can provide insights into application of AMF and the effect of AMF on other root-associated fungi in soybean, to be used as information for decision-making in field conditions.

## 2. Results

### 2.1. Plant Growth and Photosynthetic Performance

**Figure 1:**
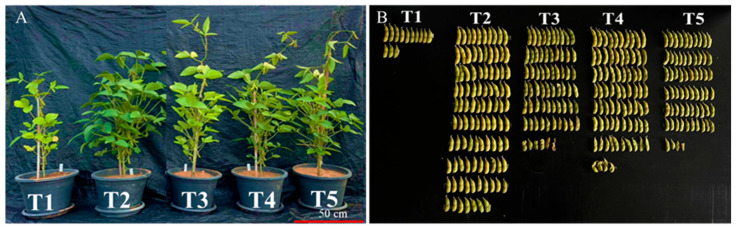
Effect of AMF inoculation on soybeans growth and yields. (**A**) Growth promotion; (**B**) pod yield. (T1: Control without mycorrhizal inoculum and synthetic fertilizer, T2: Control without mycorrhizal inoculum and with synthetic fertilizer, T3: Inoculation with *Gigaspora margarita*, T4: Inoculation with *Acaulospora dilatata*, and T5: Inoculation with *Acaulospora delicata*).

Data on plant and photosynthetic performance 60 days after germination are provided in Table 1. Inoculation with AMF increased all plant growth parameters significantly compared with the uninoculated, unfertilized plants (T1) (Figure 1A). Inoculated plants, in the absence of fertilization, were of the same size as non-inoculated plants that were fertilized. The application of synthetic fertilized resulted in plants with the largest stem diameter and highest SPAD values, which were sometimes higher than those of unfertilized, inoculated plants. Photosynthetic rate and photosynthetic water use efficiency were also increased compared with the uninoculated, unfertilized control. Transpiration rate did not differ between treatments. Plants inoculated with *A. delicata* exhibited the best photosynthetic performance, with highest rate of photosynthesis and highest photosynthetic water use efficiency.

### 2.2. Effects of AMF on Biomass, Pod Yield, and Nodules of Soybean

Table 2 shows yield performance of soybean under the different treatments. Plants inoculated with AMF, regardless of the species, had significantly higher numbers of pods and larger total seed weight, as well as larger weight of leaves, stems, and roots than uninoculated plants, unfertilized plants (Figure 1B). However, the non-inoculated, fertilized plants showed the highest pod numbers, seed weight, and weight of stems, leaves, and roots. Nodule numbers and weight exhibited a different pattern, with inoculated plants outperforming non-inoculated plants, irrespective of application of synthetic fertilizer. Leaf nutrient concentrations were lowest in the non-inoculated, not-fertilized control. Application of synthetic fertilizers resulted in the highest leaf N, P, and K concentration, with values higher than (for leaf P) or equal to those (for leaf N) of the inoculated treatments. Among the plants inoculated with AMF, nodulation was best in plants inoculated with *A. delicata*. For most other performance parameters, there were minor differences between the three AMF species, although *G. margarita* tended to be somewhat less beneficial than both species of *Acaulospora*. *Acaulospora* species are particularly effective in nutrient-poor or moderately fertile soils due to their extensive extraradical hyphal networks, which can enhance phosphorus and micronutrient uptake more efficiently than some *Gigaspora* species. Improved nutrient acquisition likely explains the higher photosynthetic rates, water use efficiency, and biomass accumulation observed in *A. delicata* inoculated plants.

### 2.3. Protein Concentration and Phytochemicals in Soybean Seeds

Total protein content, total phenolic compounds and antioxidant activity were significantly higher in the inoculated treatments than in the non-inoculated treatment, irrespective of fertilizer application. Protein content and total phenolic content were highest in plants inoculated with *G. margarita*, whereas antioxidant activity was highest in plants inoculated with *A. delicata* (Table 3).

### 2.4. Root Colonization and Spore Density

**Figure 2:**
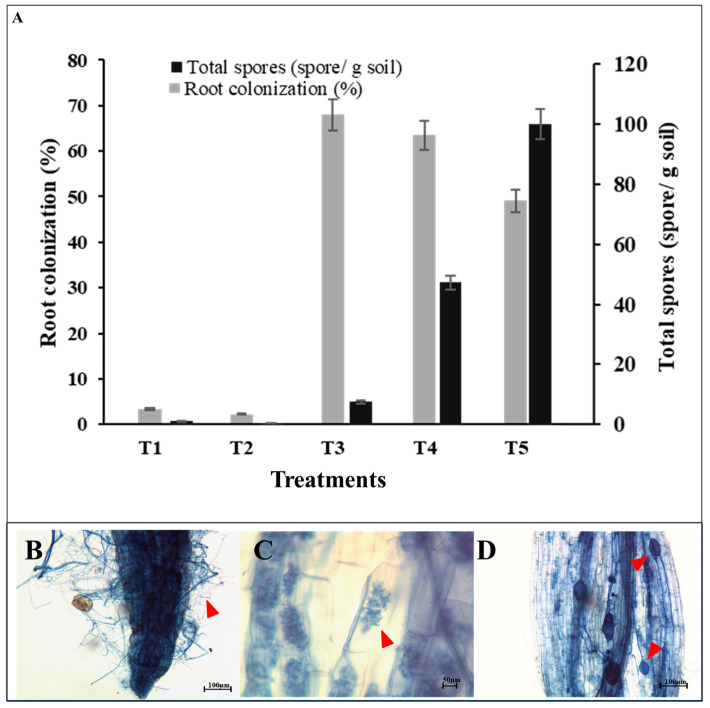
Root colonization and spore density. (**A**) Total number of AMF spores and root colonization; (**B**) hyphae around plant roots (arrowhead); (**C**) Arum-type arbuscules (arrowhead) and (**D**) vesicles (arrowhead). (T1: Control without mycorrhizal inoculum and synthetic fertilizer, T2: Control without mycorrhizal inoculum and with synthetic fertilizer, T3: Inoculation with *Gigaspora margarita*, T4: Inoculation with *Acaulospora dilatata*, and T5: Inoculation with *Acaulospora delicata*).

The non-inoculated pots, irrespective of fertilization, showed very low levels of mycorrhizal colonization (<5%). Only mycorrhizal hyphae were observed, whereas arbuscules and vesicles were absent. All inoculated plants showed good colonization, ranging from 50 to 70%, with no significant difference between the three species of AMF (Figure 2). Although total AMF colonization rates were similar among the three species, distinct species-specific colonization patterns were observed. *A. dilatata* and *A. delicata* formed all major mycorrhizal structures (hyphae, arbuscules, and vesicles), whereas *G. margarita* formed hyphae and arbuscules but lacked vesicles. In contrast, the arbuscule-dominated colonization of *G. margarita* may favor rapid nutrient transfer, consistent with the higher seed protein and phenolic contents detected in this treatment. Spore numbers showed the same pattern, with low spore numbers in the non-inoculated treatments and high spore numbers in the inoculated treatments. Spore abundance was highest in pots that were inoculated with *A. delicata*.

### 2.5. Fungal Community Analysis

**Figure 3:**
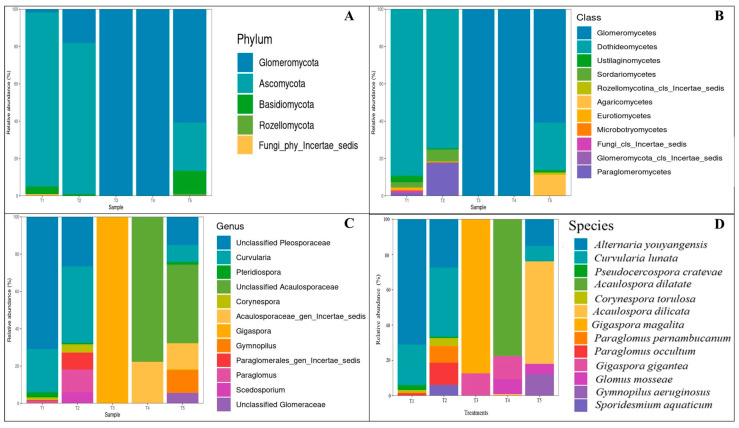
The prevalence and heat map of the ASVs identified in the samples. (**A**) The phylum level; (**B**) the class level; (**C**) the species level; and (**D**) the heat map of ASVs. (T1: Control without microbial inoculum, T2: Chemical fertilizer, T3: *Gigaspora magarita*, T4: *Acaulospora dilatata*, and T5: *Acaulospora delicata*).

The taxonomic data are visualized by barplots across all hierarchical levels (Figure 3A–D). The analysis revealed that the quantity of some pathogenic fungi increased significantly in some treatments. Nevertheless, the consistent decline in these taxa across AMF-inoculated treatments, together with reduced fungal diversity and high levels of AMF root colonization (>45%), suggests a biologically meaningful AMF-mediated filtering effect. The 30 predominant taxa were selected for further analysis at four taxonomic levels (phylum, class, genus, and species). The fungal composition of the phylum in the root samples consisted mainly of the phyla Glomeromycota, Ascomycota, Basidiomycota, Rozellomycota, Chytridiomycota, and Mucoromycota (Figure 3A). In addition, the result shows that the treatment inoculated with AMF (T3, T4 and T5) has the class of Glomeromycetes of AMF only. The most abundant fungal Amplicon Sequence Variants (ASVs) identified within the samples are illustrated in Figure 3C. The analysis of AMF species in plant roots showed that the treatment with AMF (T3, T4 and T5) revealed the presence of *G. margarita*, *A. dilatata*, and *A. delicata*, respectively (Figure 3D).

**Figure 4:**
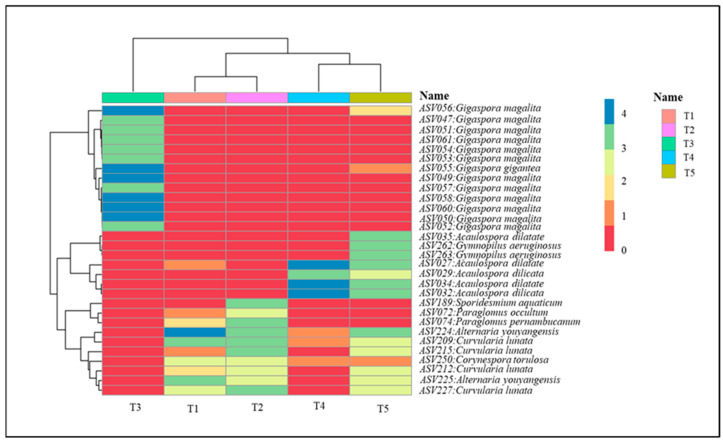
The heat map identified of the thirty most prevalent ASVs. (T1: Control without microbial inoculum, T2: Chemical fertilizer, T3: *Gigaspora magarita*, T4: *Acaulospora dilatata*, and T5: *Acaulospora delicata*).

**Figure 5:**
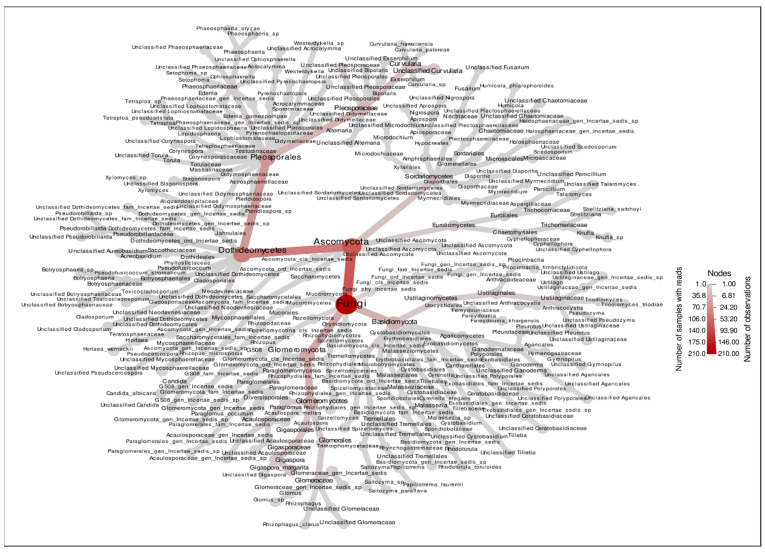
Plotting of taxa prevalence with heat tree.

The thirty most abundant ASVs identified within the samples are illustrated in Figure 4. This taxonomic variation is further illustrated in the heat map visualization, which depicts differential abundance patterns of the 30 most prevalent ASVs, with several *Gigaspora* and *Acaulospora* species exhibiting sample-specific distribution patterns. In addition, the plotting of taxa prevalence with heat tree shows the major two genus groups, namely Pleosporaceae of *Alternaria* and *Curvularia* species, which are plant pathogenic fungi and Glomeromycetes of AMF (Figure 5).

### 2.6. Alpha Diversity of Fungal Metabarcoding

**Figure 6:**
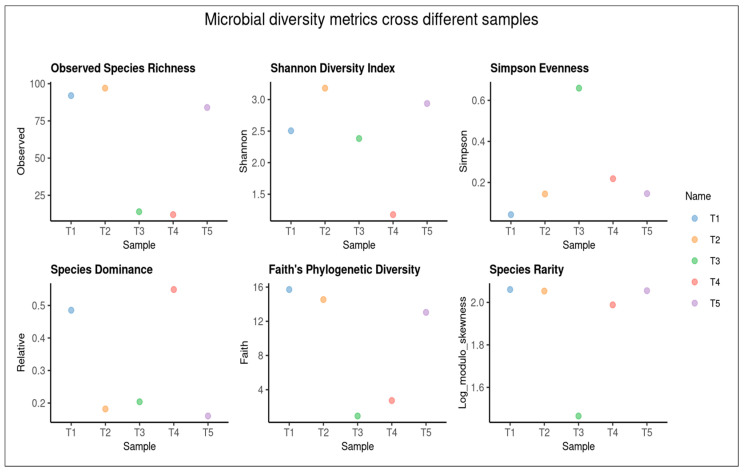
The diversity indices plots for each sample and (T1: Control without microbial inoculum, T2: Chemical fertilizer, T3: *Gigaspora magarita*, T4: *Acaulospora dilatata*, and T5: *Acaulospora delicata*).

**Figure 7:**
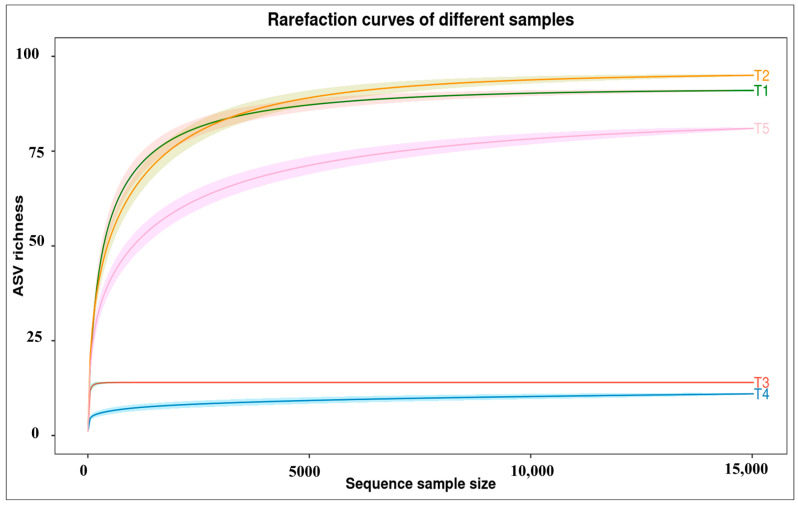
Alpha diversity rarefaction plots.

In total, 108 species of fungi from 7 classified phyla, 22 classified classes, 45 classified orders, 73 classified families, and 99 classified genera were detected. The most abundant phylum was Glomeromycota, Ascomycota, Basidiomycota, Rozellomycota, Chytridiomycota, Mucoromycota, and others. In addition, the top 13 species were *Alternaria youyangensis*, *Curvularia lunata*, *Pseudocercospora cratevae*, *Acaulospora dilatata*, *Corynespora torulosa*, *Acaulospora delicata*, *Gigaspora magarita*, *Paraglomus pernambucanum*, *Paraglomus occultum*, *Gigaspora gigantea*, *Glomus mosseae*, *Gymnopilus aeruginosus*, and *Sporidesmium aquaticum*. Alpha diversity analysis was conducted using characterizing within-sample diversity, defined as the taxonomic richness and evenness observed in individual samples. This standardization technique enables valid cross-sample comparison of diversity metrics by simulating uniform sampling effort across all specimens, thereby providing robust estimates of expected total diversity (Figure 6). Diversity indices are statistical measures used to quantify the diversity of a given community or ecosystem. These indices were calculated using the ggpubr package in R software version 2.15.1 (Figure 7).

## 3. Discussion

In this study, we demonstrated that in the absence of fertilizer application, AMF inoculation resulted in larger plants, higher photosynthesis and photosynthetic water use efficiency, more biomass, and higher leaf nutrient concentrations. They also contained higher amounts of protein and phenolic compounds and exhibited higher antioxidant activity. Application of fertilizer in the absence of inoculation resulted in the highest plants with the largest biomass, and higher photosynthesis and photosynthetic water use efficiency than inoculated plants. The results of our study are consistent with previous research by Tavares et al. [12], who reported that soybean plants submitted to water restriction inoculated with *G. margarita* and *G. gigantea* can promote growth and photosynthesis. In another study, soybean inoculated with *Glomus* sp. and *Gigaspora* sp. promoted growth and biomass [13]. In addition, our results agree with previous reports on other plant species. Agustin et al. [14] reported that the effects of inoculation with *Acaulospora* sp., *A. tuberculata*, *Entrophospora* sp., and *Gigaspora* sp. on growth performance of *Eucalyptus pellita* and *Acacia crassicarpa* showed the highest impact on plant height, stem, and diameter. The observed AMF-induced improvements in soybean growth and seed quality are likely mediated by underlying molecular and hormonal mechanisms. Recent transcriptomic evidence indicates that soybean reproductive development is tightly regulated by coordinated hormone signaling networks, particularly involving auxin, cytokinin, gibberellin, and abscisic acid pathways, which control carbon allocation, seed filling, and protein accumulation [15]. Arbuscular mycorrhizal fungi are known to modulate host hormone homeostasis and gene expression related to nutrient transport, stress signaling, and secondary metabolism, thereby indirectly influencing seed composition and phytochemical accumulation [16]. Therefore, the enhanced protein content, phenolic compounds, and antioxidant activity observed in AMF-inoculated soybean may reflect AMF-driven reprogramming of hormone-regulated transcriptional networks during seed development, as supported by recent omics-based studies [15].

Mycorrhizal inoculation significantly increased pod weight and seed weight. Especially, inoculation with *A. dilatata* resulted in significantly higher pod, 100-seed weight, biomass relative to no inoculation and no synthetic fertilizer. Similar data were shown previously. Soybean inoculated with *Glomus* sp. and *Gigaspora* sp. showed the maximum number of seeds per plant, weight of seeds per plant, and weight of 100 seeds compared to control [13]. Mycorrhizal inoculation of soybean had a distinct effect on functional compounds in seeds, and this effect could not be mimicked by the application of synthetic fertilizer. Among the mycorrhizal treatments, plants inoculated with *A. delicata* generally performed best, although plants inoculated with *G. margarita* possessed the highest amounts of protein phenolic compounds and antioxidant activity. Although the biochemical mechanisms were not directly investigated in this study, the increase in phenolic compounds and antioxidant activity in AMF-inoculated plants can be explained by known AMF-induced metabolic responses. AMF colonization is known to activate plant signaling pathways and stimulate secondary metabolism, particularly the phenylpropanoid pathway, which is responsible for phenolic and antioxidant compound biosynthesis [16]. In addition, improved phosphorus and nitrogen uptake in AMF-colonized plants provides the metabolic resources required for secondary metabolite production. AMF can also induce a priming effect that enhances antioxidant defense systems, leading to higher antioxidant capacity in plant tissues [17,18]. Although not directly measured, these well-established mechanisms provide a plausible explanation for the observed increase in phytochemicals. Interestingly, the number and weight of nodules were significantly higher in inoculated plants than in non-inoculated plants that were fertilized. We cannot exclude that this difference was caused by the nature of the synthetic fertilizer applied that contained both N (that potentially repressed nodule formation) and P (that potentially stimulated nodule formation). AMF stimulates rhizobia through enhanced plant P uptake [19]. An increase in root nodule formation enhances nitrogen availability to plant, resulting in superior plant height [13]. Increasing nodulation and N_2_ fixation usually increases the number of leaves, plant height, and stem diameter of legumes. Ngosong et al. [20] reported that the soybean inoculation with plant growth-promoting bacteria (PGPB) and AMF increased the number of root nodules by 67% and 57%, respectively, whereas co-application of PGPB and AMF increased the number of root nodules by 68%, indicating lack of synergistic effects.

Soybean inoculation with AMF resulted in plants with highest protein concentration and phytochemicals in soybean seeds. Especially, *G. magarita* increased total protein content and total phenolic compounds compared to both the fertilized und unfertilized controls. For antioxidant activity inoculation with *A. delicata* was superior. Marro et al. [21] reported similar effects, with soybean inoculated with *A. scrobiculata* significantly increased seed protein content (12–14%) compared with soybean grown without AMF inoculation. Differential effects of fertilizer application and AMF inoculation by different species of AMF are novel and potentially relevant, because the nutritional quality of soybean used in healthy-food markets is primarily based on phytochemical composition and protein content.

The metabarcoding analysis performed in this study exhibited the presence of different fungal species in the plant. While these analyses have primarily focused on the microbial communities inhabiting the root, the AMF diversity in plant roots has been examined. The analysis of ITS amplicon sequence data across five samples (T1–T5) reveals significant patterns in fungal community structure and diversity. The particular emphasis on the genus *Curvularia* and *Alternaria*, which is a genus that can cause soybean diseases [22] and pathogens in cucurbit production [23], is found in treatment uninoculated with AMF. *Acaulospora* and *Gigaspora* are arbuscular mycorrhizal fungi that form symbiotic relationships with plants [12,24]. Taxonomic profiling demonstrates considerable heterogeneity in microbial composition among samples. Glomeromycota dominated the majority of samples, with the treatments of T1, T2, and T5 exhibiting substantial abundance of Ascomycota, Basidiomycota, Rozellomycota, Chytidiomycota, and Mucoromycota. In contrast, the treatment T3 and T4 displayed a distinct taxonomic profile characterized by the predominance of *Acaulospora* and *Gigaspora* at the genus level. This taxonomic variation is further illustrated in the heat map visualization, which depicts differential abundance patterns of the 30 most prevalent ASVs, with several *Curvularia* species exhibiting sample-specific distribution patterns (T1, T2, and T5). In microscale environments, pathogenic fungi in plants are likely to be dominated by AMF interactions that further affect the growth of host plants [25], and reduce the infection due to inferior competition of pathogenic fungi [26].

Differences in yield, seed composition, and pathogen suppression among AMF treatments may be caused by species-specific colonization patterns. The results are consistent with the root colonization of AMF in more than 45% of the soybean roots. *Acaulospora dilatata* and *A. delicata* formed complete mycorrhizal structures, including arbuscules and vesicles. These differences are functionally relevant because arbuscules are the primary sites of nutrient exchange, while vesicles contribute to carbon storage and symbiotic persistence. The presence of vesicles in *Acaulospora*-inoculated roots may support more stable and sustained nutrient acquisition, potentially explaining the superior growth promotion and antioxidant activity observed with *A. delicata*. Since soil and environmental conditions were kept constant across treatments, the observed shifts in fungal community structure are most likely driven by biotic interactions, including competitive exclusion by AMF, changes in root exudation patterns, improved plant nutritional status, and AMF-induced activation of plant defense mechanisms. In contrast, *Gigaspora margarita* formed arbuscules but lacked vesicles, suggesting a more transient symbiosis that may support rapid nutrient transfer but reduced persistence within roots. These structural differences likely influence host nutrient allocation, seed protein accumulation, and secondary metabolism. In addition, AMF are known to modulate plant defense pathways by priming systemic resistance, enhancing jasmonic acid- and salicylic acid-dependent signaling, and activating the phenylpropanoid pathway [17,18]. This phenomenon is in line with previous studies reporting that extensive AMF colonization can effectively suppress root pathogens by occupying infection sites and enhancing plant defense mechanisms [16,27]. Moreover, AMF symbiosis is known to improve host plant immunity through the activation of systemic resistance pathways and the production of antifungal metabolites within the rhizosphere and root cortical cells. [28]. Analyses of alpha diversity (Figure 7) indicate marked differences in community complexity. Treatment T1 and T2 exhibited elevated species richness (92 and 97 observed species, respectively) and phylogenetic diversity (Faith’s PD of 15.71 and 14.54), indicating complex fungal assemblages. Conversely, treatments T3 and T4 demonstrated markedly reduced diversity (14 and 12 observed species), suggesting communities subject to stronger AMF filtering or selection pressures. In root-associated microbial communities, reduced diversity can reflect strong biotic filtering imposed by dominant symbionts such as AMF, leading to the selective exclusion of competing or potentially pathogenic taxa [29]. However, such reductions may also decrease functional redundancy and ecosystem resilience, potentially increasing vulnerability to environmental perturbations or secondary pathogen invasion over longer timescales [30,31]. In this study, lower fungal diversity coincided with high levels of AMF root colonization, improved plant growth, and reduced abundance of putative pathogenic fungi, suggesting that the observed decline likely represents effective microbial filtering rather than detrimental community disruption. Nevertheless, because the experiment was short-term and focused primarily on plant growth and yield responses, potential negative consequences of reduced microbial diversity on long-term soil health and system stability cannot be excluded. Similarly, Sarathambal et al. [32] report that the AMF inoculated in ginger (*Zingiber officinale* Rosc.) demonstrated 50% reduction in disease incidence when challenged with *P. myriotylum*. In addition, pepper plants (*Capsicum annuum* L.) cultivation with *Funneliformis mosseae*, *Rhizophagus intraradices*, and *Claroideoglomus etunicatum* had significantly reduced disease severity, with disease inhibition (DI) reaching up to 58%, depending on the *Fusarium solani* and *Fusarium* sp. pathogen [33]. Shannon diversity indices further corroborate these patterns, with T2 displaying the highest diversity value (3.18) and T4 the lowest (1.18). Rarefaction analyses (Figure 7) indicate sufficient sequencing depth for the majority of samples, as evidenced by asymptotic curve behavior, particularly for treatments T1, T2, and T5. The lower plateaus observed for treatments T3 and T4 confirm genuinely depauperate communities rather than artifacts of insufficient sampling depth. Concerning *Curvularia* specifically, this genus constitutes a significant component of the mycobiome in samples of treatments T1, T2, and T5, as demonstrated by genus-level prevalence data (Figure 3C), with relative abundances ranging from approximately 10–15%. The distribution patterns suggest the influence of AMF selection processes and potential dispersal mechanisms on community composition on AMF infection. This decrease in disease can be linked to several biochemical mechanisms, including the increased activity of defense-related enzymes as well as an elevation in phenolic content induced by AMF colonization. These changes strengthen the host plant protective mechanisms against pathogen invasion [32].

## 4. Materials and Methods

### 4.1. AMF Preparation

The AMF strains used in this study were *Gigaspora margarita*, *Acaulospora dilatata*, and *A. delicata*, obtained from the Mycorrhizal and Fungal Technology Laboratory, Department of Microbiology, Faculty of Science, Khon Kaen University, Thailand. AMF spore propagation was carried out by a pot culture technique using maize (*Zea mays* L.) as host plant as described by Boonlue et al. [7]. Briefly, soil was sterilized twice (using an autoclave at 121 °C for 2 h) before filling in 20 cm diameter plastic pots. Maize seeds were surface-sterilized by soaking in 6% sodium hypochlorite solution for 3 min and subsequently added into the soil in these plastic pots. Approximately 200 fungal spores were inoculated per each pot. The maize was grown in a greenhouse at 30–35 °C with daily irrigation using tap water. After 90 days, irrigation was stopped to allow the plants to dry for 5 days [8]. After that, the plants were cut off at a position just above the (dried) soil surface. Finally, dried soil was crushed into finely ground particles. The purity of AMF spores and the total number of spores were determined using the sucrose centrifugation method [34]. Dried soils containing AMF spore, mycelia, and colonized root pieces were then used as soil inoculum in the experiment.

### 4.2. Soil Preparation

Properties of the sandy loam soil used in this study were as follows: pH 5.9, electrical conductivity (EC) 0.14 dS/m, organic matter (OM) content 6.4 g kg^−1^, available nitrogen (N) content of 0.060 mg kg^−1^, total phosphorus (P) content 145 mg kg^−1^, available P content 22.7 mg kg^−1^, potassium (K) content 88 mg kg^−1^, exchangeable K content 50.2 mg kg^−1^, calcium (Ca) content 790 mg kg^−1^, sodium (Na) content 29 mg kg^−1^, and magnesium (Mg) content 68 mg kg^−1^. Prior to use, rocks, wood chips, and plant debris were removed.

### 4.3. Soybean Preparation

Seeds of *Glycine max* cultivar Morkhor 60 were kindly provided by Assist. Prof. Dr. Sompong Chankaew, Department of Agronomy, Faculty of Agriculture, Khon Kaen University, and Plant Breeding Research Center for Sustainable Agriculture, Khon Kaen University, Khon Kaen provide, Thailand.

### 4.4. Experimental Design

The experiment was conducted in an enclosed greenhouse at the Department of Agronomy, Faculty of Agriculture, Khon Kaen University, Khon Kaen, Thailand (16°28′27.7″ N, 102°48′36.5″ E; 190 m above sea level) during the rainy season (September–November 2024). The climate consists of an average temperature of 18–30 °C, partly cloudy with a rate of 63%, 40–45 mm of rainfall, 15–20% of precipitation, 70–80% moisture, 12 h of sunlight, and a wind speed of 11.2 km/h. The experiment was set up according to a completely randomized design (CRD), consisting of five treatments, each replicated nine times, for a total of 45 pots. These treatments were T1: a control without mycorrhizal inoculum addition or synthetic fertilizer, T2: a control without mycorrhizal inoculum addition and with synthetic fertilizer (Nitrogen (N)-Phosphorus (P)-Potassium (K), 15-15-15 at 2 g/pot), T3: plants inoculated with *G. margarita*, T4: plants inoculated with *A. dilatata*, and T5: plants inoculated with *A. delicata*. The soils were then filled into 40 cm top-diameter pots for cultivation of soybean seedlings. The *G. max* was grown in a greenhouse with daily irrigation using tap water.

### 4.5. Plant Performance

Plant height, stem diameter, SPAD, and photosynthesis were measured at reproductive stage 4.5 (R4.5: full pod and first beginning seed at 60 days after germination). The height of soybean plants was measured by a standard stick method. Diameter of soybean stem was measured at 2.5 cm above the ground using a vernier caliper (Mitutoyo, Kanagawa, Japan). Greenness of leaves was determined from the second expanded leaf from the top of the main stem by chlorophyll meter SPAD-502 plus (KONICA MINOLTA, Tokyo, Japan). The second expanded leaves from the top were analyzed for photosynthesis rate, stomatal conductance, and transpiration rate using a LI-6400XT portable photosynthesis system (LI-COR Bioscience, Lincoln, NE, USA). Photosynthetic water use efficiency (WUE) was calculated from photosynthetic rate divided by transpiration rate.

Leaves, stems, roots, number of pods, and total seed weight, and number of nodules were measured at reproductive stage R8 at 90 days after germination (R8: full maturity, 95% of pods have reached mature pod color). The number of root nodules was counted. Dried stem, leaf, and root samples were ground into powder and used for nutrient uptake analysis by determining total N and P concentrations. N concentration was determined by the micro-Kjeldahl method [35] and indophenol blue [36]. P concentration was determined using the wet oxidation method with HNO_3_:HClO_4_ (2:1, *v*/*v*). Then, the color intensity was measured at a wavelength of 420 nm. K content was detected using a flame photometer at 768 nm (flame photometer, Model 410 Sherwood, Cambridge, UK) [37]. The total uptake of N, P, and K was calculated multiplying dry biomass by N or P concentration.

### 4.6. Phytochemical Analysis of Soybean Seeds

Dried seeds were finely ground, and an amount of 1.0 g was extracted with 10 mL of methanol, shaken for 2 h, and then centrifuged at 6000 rpm for 10 min at 4 °C. The mixture was filtered (Whatman No. 1 filter paper, Cytiva, Marlborough, MA, USA). The supernatants were stored at −40 °C in the dark until analysis. 2,2-diphenyl-1-picrylhydrazyl (DPPH) free radical scavenging activity was determined according to the method described by Leong and Shui [33], with some modifications. Freshly prepared solution of 0.1 mM solution of DPPH in methanol was prepared with absorbance 517 nm. An aliquot of 100 µL of each sample (with appropriate dilution) was mixed with 4.0 mL of DPPH solution, then allowed to stand at room temperature for 30 min before measurement. The percentage of radical scavenging ability was calculated by using the following formula:

Scavenging ability (%) = (Absorbance 515 nm of control) − (Absorbance 515 nm of sample)/(Absorbance 515 nm of control) × 100

Total phenolic content was determined using the method of Nacoon et al. [19] based on 125 µL of extracted samples and 250 µL Folin-Ciocalteu’s reagent, followed by the addition of 3 mL distilled water. The solution was mixed well and then allowed to stand for 6 min, after which 2.5 mL of 7% sodium carbonate (Na_2_CO_3_) was added. The reaction mixture was allowed to stand for 90 min at room temperature before measuring the absorbance at 760 nm (Hitachi High-Tech Science Corporation, Tokyo, Japan). Gallic acid was used as a calibration standard, and the results were expressed as mg gallic acid equivalent per 100 g sample.

### 4.7. Total Protein Content

Total protein content was determined using the method of Bello et al. [38]. Soybean powder and alkaline solution (0.5% NH_4_OH) were mixed in a ratio of 1:10 (*w*/*v*), and the suspension was constantly mixed (150 rpm) at 52 °C for 12 h. The mixture was centrifuged for 5 min, at 4 °C at 10,000 rpm. Then, the pellet was discarded, and the supernatant was adjusted to pH 4.5 using a solution of 7.5% HCl and centrifuged, as mentioned before. The pellet was washed with distilled water. Next, the pellet was dried at 60 °C for 24 h, and thereafter the protein content was determined by the Lowry method [39].

### 4.8. Determination of Root Colonization and Total Number of AMF Spores

Root samples were randomly collected to check for AMF root colonization intensity. Briefly, soil adjacent to fresh roots was washed off with tap water. Roots were cleared with 10% KOH for 5 min at 95 °C and then acidified in 2% HCl overnight. For microscopic visualization, root samples were stained with 0.05% trypan blue solution [40] before cutting into pieces with a length of 1.0 cm. Percentage of AMF colonization was determined according to the method described by Trouvelot et al. [41]. Cellular structures of AMF including vesicles, arbuscules, and hyphae were observed using a microscope at 40× magnification (SMZ745T Nikon, Tokyo, Japan).

Total number of AMF spores in each treatment, soil samples from pots were randomly collected for analysis using the sucrose centrifugation method [30]. Five grams of soil samples were suspended with distilled water and then centrifuged at 4000 rpm for 5 min to retrieve supernatant. The supernatant was mixed with 50% sucrose solution and then centrifuged at 3000 rpm for 2 min. The supernatant containing fungal spores was sieved through a 45 µm sieve. Fungal spores left on the sieve were rinsed with distilled water to remove sucrose solution. The spores were then placed on a filter paper and transferred into a Petri dish for counting under a three-dimensional microscope (Stereoscopic microscope, Nikon model L C-LEDS, Tokyo, Japan). The number of spores in each sample was counted in 4 repetitions according to the method.

### 4.9. Fungal Community Analysis

#### 4.9.1. Extraction of DNA and Sequencing

Root samples were randomly collected to identify fungi and assess microbial diversity after Illumina sequencing. In total, 100 mg of root samples were crushed in a mortar and pestle with liquid nitrogen to extract total genomic DNA with the plant genomic DNA kit (TIANGEN, Beijing, China), following the manufacturer’s instructions. The concentration of extracted DNA was measured using a NanoDrop^®^ ND-1000 spectrophotometer device (Thermo Scientific, Whaltam, MA, USA).

Library preparation and DNA sequencing tasks were conducted at U2bio (Thailand) Co., Ltd. (Bangkok, Thailand). The ITS2 region (forward, 5′-GCATCGATGAAGAACGCAGC-3′ and reverse, 5′-TCCTCCGCTTATTGATATGC-3′) was selected as the target amplicon for the metabarcoding study of the fungal community. All procedures were performed using one microgram of DNA per sample. Initial quality control (QC) included quantification, integrity evaluation, and purity assessment of DNA using the Agilent 5400 system (Santa Clara, CA, USA). DNA was then fragmented by sonication, after which the fragments underwent end polishing, phosphorylation, and A-tailing reactions. The processed DNA fragments were ligated with Illumina adapters and amplified via polymerase chain reaction (PCR). Library preparation was conducted using the Illumina Ultra DNA Library Prep Kit (NEB, Ipswich, MA, USA), followed by removal of PCR primers. A size-selection step was performed to obtain fragments with an average length of 550 base pairs (bp). Library quantification was carried out using both Qubit fluorometry and real-time PCR. The size distribution of the libraries was subsequently analyzed with a bioanalyzer as an additional QC step. Pooled libraries were sequenced on the Illumina NovaSeq™ 6000 platform (San Diego, CA, USA).

#### 4.9.2. Bioinformatics

After sequencing, the generated raw data were demultiplexed using QIIME2 [42] for removal of barcode sequences and the separation of samples. Following amplification, sequence data underwent noise reduction via a galaxy workflow using DADA2 (Galaxy Version 1.20 + galaxy0) [43], enhancing precision for subsequent analyses and biological interpretation. Data normalization was performed using the scaling with ranked subsampling (SRS) methodology [44], with corresponding parameters and curves. Taxonomic classification was conducted utilizing a BLAST classifier software version 2.16.0 [45] trained on taxonomically defined reference sequences from UNITE database version 10.0 [46]. Taxa prevalence was analyzed using the phyloseq package [47] in R. This analysis evaluates the distribution and frequency of specific taxonomic levels within the mycobiome dataset by quantifying taxon presence across multiple samples and calculating their occurrence proportions.

### 4.10. Statistical Analysis

Data were analyzed using Statistix 10 software. One-way analysis of variance (ANOVA) was performed to analyze differences among the means of the data. Fisher’s Least Significant Difference (LSD) was applied to evaluate significant differences at a 95% confidence interval (*p* ≤ 0.05).

## 5. Conclusions

The application of AMF inoculum enhanced the protein content and phytochemical accumulation in soybean, providing greater benefits than conventional fertilizer application. When seed quality is prioritized over yield quantity, mycorrhizal management may serve as a more effective and sustainable strategy than chemical fertilization. Interestingly, metabarcoding analysis revealed that soybean cultivation with AMF also reduced root colonization by pathogenic fungi in non-sterile soil, highlighting the potential of AMF to improve both plant quality and soil health.

## Figures and Tables

**Table 1:** Plant growth parameters and photosynthetic performance 60 days after transplanting.

Treatments	Height (cm)	Diameter (mm)	SPAD	Photosynthetic Rate (µmol CO_2_ m^−2^ s^−1^)	Stomatal Conductance (mol H_2_O m^−2^ s^−1^)	Transpiration Rate (mmol H_2_O m^−2^ s^−1^)	Water Use Efficiency (µmol CO_2_/mmol H_2_O m^−2^ s^−1^)
T1	74.0 b	3.66 d	30.6 c	8.26 d	0.45 aa	1.66 a	5.78 c
T2	105.3 a	10.73 a	41.6 a	21.03 a	0.49 a	1.85 a	11.90 ac
T3	95.0 ab	9.06 ab	37.0 ab	15.31 b	0.502 a	2.36 a	7.20 bc
T4	115.0 a	8.10 bc	36.6 abc	11.74 c	0.53 a	2.12 a	5.86 bc
T5	112.3 a	6.83 c	32.2 cb	18.85 a	0.39 a	1.73 a	10.87 a
%CV.	12	14	10	12	28	30	29
F-test	**	**	*	**	ns	ns	*

Numbers followed by the same letters in each column indicate data that are not significantly different according to LSD test. ns, non-significant difference; * Significant difference at *p* ≤ 0.05, ** Significant difference at *p* ≤ 0.01; (T1: Control without mycorrhizal inoculum and synthetic fertilizer, T2: Control without mycorrhizal inoculum and with synthetic fertilizer, T3: Inoculation with *Gigaspora margarita*, T4: Inoculation with *Acaulospora dilatata*, and T5: Inoculation with *Acaulospora delicata*).

**Table 2:** Pod yield, plant biomass, nodule, and nutrient uptake 90 days after germination.

Treatments	Number of Pods	Total Seed Weight (g Plant^−1^)	100 Seed Weight (g)	Biomass (g Plant^−1^)	Number of Nodules Plant^−1^	Nodules Fresh Weight (g)	Total N (mg g^−1^)	Total P (mg g^−1^)	Total K (mg g^−1^)
T1	13.7 d	5.1 c	10.3 c	10.5 c	1.7 b	0.20	1.07 b	0.80 c	4.20 b
T2	97.0 a	30.0 a	12.6 ab	63.8 a	4.7 b	0.8 d	8.00 a	2.80 a	8.70 a
T3	66.3 c	15.1 b	10.6 bc	32.9 b	8.3 b	1.9 c	6.80 a	1.40 b	6.60 ab
T4	74.0 b	14.06 b	13.07 a	36.5 b	9.7 b	2.2 b	5.90 a	1.00 b	7.30 a
T5	64.3 c	14.5 b	13.1 a	34.2 b	22.3 a	5.8 a	6.90 a	1.40 b	6.90 ab
%CV.	5	12	10	16	24	14	18	12	11
F-test	**	**	*	**	**	**	**	**	ns

Numbers followed by the same letters in each column indicate data that are not significantly different according to LSD test. ns, non-significant difference; * Significant difference at *p* ≤ 0.05, ** Significant difference at *p* ≤ 0.01; (T1: Control without mycorrhizal inoculum and synthetic fertilizer, T2: Control without mycorrhizal inoculum and with synthetic fertilizer, T3: Inoculation with *Gigaspora margarita*, T4: Inoculation with *Acaulospora dilatata*, and T5: Inoculation with *Acaulospora delicata*).

**Table 3:** Total protein content, total phenolic compounds content, and antioxidant activity of soybean seeds in the various treatments.

Treatments	Total Protein (mg g^−1^)	Total Phenolic Compound (mg Gallic eq 100 g^−1^ DW)	Antioxidant (% Radical Scavenging)
T1	452 c	126.1 bc	29.3 c
T2	401 d	112.3 c	44.4 bc
T3	522 a	196.5 a	33.1 bc
T4	494 b	175.3 a	60.2 ab
T5	496 b	167.0 ab	72.1 a
%CV.	23	15	25
F-test	**	**	*

Numbers followed by the same letters in each column indicate data that are not significantly different according to LSD test, * Significant difference at *p* ≤ 0.05, ** Significant difference at *p* ≤ 0.01; (T1: Control without mycorrhizal inoculum and synthetic fertilizer, T2: Control without mycorrhizal inoculum and with synthetic fertilizer, T3: Inoculation with *Gigaspora margarita*, T4: Inoculation with *Acaulospora dilatata*, and T5: Inoculation with *Acaulospora delicata*).

## Data Availability

No, I do not have any research data outside the submitted manuscript file.
